# Dissociation of natriuresis and diuresis by oxytocin molecular forms in rats

**DOI:** 10.1371/journal.pone.0219205

**Published:** 2019-07-03

**Authors:** Marek Jankowski, Bogdan A. Danalache, Eric Plante, Ahmed Menaouar, Maria Florian, Ju Jing Tan, Ryszard Grygorczyk, Tom L. Broderick, Jolanta Gutkowska

**Affiliations:** 1 Department of Medicine, University of Montreal, CRCHUM, Quebec, Canada; 2 Department of Physiology, College of Graduate Studies, Midwestern University, Glendale, AZ, United States of America; Indiana University School of Medicine, UNITED STATES

## Abstract

In the rat, oxytocin (OT) produces dose-dependent diuretic and natriuretic responses. Post-translational enzymatic conversion of the OT biosynthetic precursor forms both mature and C-terminally extended peptides. The plasma concentrations of these C-terminally extended peptides (OT-G; OT-GK and OT-GKR) are elevated in newborns and pregnant rats. Intravenous injection of OT-GKR to rats inhibits diuresis, whereas injection of amidated OT stimulates diuresis. Since OT and OT-GKR show different effects on the urine flow, we investigated whether OT-GKR modulates renal action by inhibition of the arginine-vasopressin (AVP) receptor V_2_ (V_2_R), the receptor involved in renal water reabsorption. Experiments were carried out in the 8-week-old Wistar rats receiving intravenous (iv) injections of vehicle, OT, OT-GKR or OT+OT-GKR combination. OT (10 μmol/kg) increased urine outflow by 40% (*P*<0.01) and sodium excretion by 47% (*P*<0.01). Treatment with OT-GKR (10 μmol/kg) decreased diuresis by 50% (*P*<0.001), decreased sodium excretion by 50% (*P*<0.05) and lowered potassium by 42% (*P*<0.05). OT antagonist (OTA) reduced diuresis and natriuresis exerted by OT, whereas the anti-diuretic effect of OT-GKR was unaffected by OTA. The treatment with V_2_R antagonist (V_2_A) in the presence and absence of OT induced diuresis, sodium and potassium outflow. V_2_A in the presence of OT-GKR only partially increased diuresis and natriuresis. Autoradiography and molecular docking analysis showed potent binding of OT-GKR to V_2_R. Finally, the release of cAMP from CHO cells overexpressing V_2_ receptor was induced by low concentration of AVP (EC50:4.2e-011), at higher concentrations of OT (EC50:3.2e-010) and by the highest concentrations of OT-GKR (EC50:1.1e-006). OT-GKR potentiated cAMP release when combined with AVP, but blocked cAMP release when combined with OT. These results suggest that OT-GKR by competing for the OT renal receptor (OTR) and binding to V_2_R in the kidney, induces anti-diuretic, anti-natriuretic, and anti-kaliuretic effects.

## Introduction

It is well-established that oxytocin (OT) plays a critical role in reproductive function, including parturition and lactation [1]. OT is also implicated in the regulation of water and electrolyte homeostasis and cardiovascular function [2]. OT and arginine vasopressin (AVP, antidiuretic hormone) are nonapeptides synthesized by the hypothalamus and although these neuropeptides are structurally similar in amino acid composition and sequence, they fundamentally act on different receptors for physiological function [1, 3]. Only one receptor for OT is currently known (OTR) whereas three receptor subtypes (V_1a_, V_1b_ and V_2_) have been identified for AVP[1]. In the rat kidney, specific [^3^H]OT binding to glomeruli was demonstrated by autoradiography and the presence of OTR on glomerular structures may suggest the involvement of OT on renal hemodynamics, including the effects primarily attributed to AVP [4]. The distribution of OTR undergo significant reshaping during the postnatal development, leading to an exclusive cortical location in the adult [4]. Although OT and AVP have a higher affinity for their own receptors, their selectivity is not absolute and significant crosstalk occurs with OT and AVP receptors [1]. Depending on the concentration and route of administration, OT evokes both diuretic and antidiuretic responses in the rat [5]. At physiological concentrations, OT induces diuresis and natriuresis in rodents [6] by mechanisms that include enhanced ANP secretion from the heart and increased production of nitric oxide (NO) by the kidneys [7, 8]. At pharmacological concentrations [9, 10] or in the response to chronic OT infusions, the antidiuretic effect of OT has been suggested to occur by binding to the V_2_ receptor (V_2_R).

OT is primarily synthesized in the hypothalamus as a precursor peptide. In the initial steps of biosynthesis of this hormone, OT is linked to the tripeptide Gly-Lys-Arg (GKR) and neurophysin I. During post-translational processing, proteolytic cleavage releases neurophysin I from the precursor peptide generating carboxy-extended prohormones, including OT-GKR, OT-GK and OT-G which are then finally converted into mature amidated OT [11]. Plasma OT-GKR levels are low in postmenopausal women and rhesus monkeys and increased with estrogen therapy, resulting in reductions in blood pressure and systemic vascular resistance [12, 13]. In rat uterus, extended forms of OT increase during gestation and their synthesis is reduced by administration estrogen and progesterone antagonists [14]. The process of initial translation product through amidated OT is developmentally regulated [14] and high concentrations of OT-GKR, OT-GK and OT-G have been detected in the developing brain [11], heart [15] and foetal plasma [16]. Changes in the relationship between extended forms of OT and mature amidated OT in umbilical arterial and venous plasma take place with labor, preceding the dramatic changes of neonatal diuresis and natriuresis. In this study, we hypothesize that OT-GKR is involved in these changes and targets the receptors present in the kidney. To understand mechanism of action of OT-GKR on receptors in kidney, we first investigated the combined OT and OT-GKR effects on water and electrolyte homeostasis in the rat, followed by an analysis of molecular docking of OT-GKR into V_2_R and OTR molecular models. Finally, we analyzed the effects of OT and OT-GKR on the cAMP release from CHO cells expressing transfected V_2_R.

## Material and methods

### *In vivo* study design

Experiments were performed using 8-week-old Wistar rats (225-250g) purchased from Charles River laboratories (St. Constant, QC, CA). Rats were housed in a standard temperature and light condition with access to water and irradiated standard rodent diet (Harlan Laboratories Inc., IN, USA) *ad libitum*. At the end of experiments, rats were anaesthetized with sodium pentobarbital (5mg/100 g body wt. i.p.; 20 𝜇mol/100 g body wt.) and blood samples (2mL, EDTA) were collected. Rats were euthanized by cervical decapitation. All protocols were approved by the Institutional Bioethics Committee of CRCHUM (le Comité institutionnel de protection des animaux du CHUM, protocol H08003jGr) in agreement with the recommendations of guidelines of the Canadian Council on Animal Care.

To determine the dose effect of OT and OT-GKR on urine water and electrolyte excretion, rats (n = 24) received intravenous (iv) injections of OT (Sigma-Aldrich, St. Louis, MO, USA) and OT-GKR (GenScript Corp., Piscataway, NJ, USA) at 4 different doses (1, 2, 4 and 10 μmol/kg) or with vehicle (0.9% sodium chloride, Baxter, Mississauga, ON, CA). Peptides were dissolved at the desired concentration in 0.3 mL of the vehicle and administered as a single bolus in the tail vein. Injections of OT and OT-GKR were performed in random order in the same rats separated by a 2–3 day interval.

A second set of experiments was performed to compare the renal effects of the highest doses (10 μmol/kg) of OT and OT-GKR in combination with a specific antagonist of OTR (OTA; [d(CH2)5, Tyr(Me)2, Thr4, Orn8, Tyr-NH29]-vasotocin, Bachem Americas, Torrance, CA, USA) and V_2_R (V_2_A; [d(CH2)5, D-Ile2, Ile4, Tyr-NH29]-AVP), generous gift from Dr. Gainer, NINDS, NIH). Both antagonists (OTA, V_2_A) were administered to rats at the concentration of 10 μmol/kg under normal conditions (0.3 mL iv injection) and with blood volume expansion (5 mL iv injection). Rats (n = 24 per condition) received combination treatment in random order separated by a 2–3 day interval. For both series of experiments, rats were placed in metabolic cages for a period of 5 hours to collect urine for the determination of sodium and potassium levels. Metabolic cages were kept in a bright and quiet room and rats were deprived of food and water during this period.

### Urine analysis

The concentrations of excreted sodium and potassium were determined by flame spectrometry (iCE 3000 series AA spectrometer; Thermo Fisher Scientific, Cambridge, UK).

### Fura-2-based calcium measurements in H9c2 cells

To detect potential difference in cytosolic Ca^2+^ concentration upon the treatment with OT and OT-GKR the experiments were performed on rat cardiac H9c2 cell line obtained from American Type Culture Collection (Rockville, MD, USA). Cells were grown on Dulbecco minimal essential medium (DMEM) purchased from Invitrogen Corporation (Grand Island, NE, USA) and supplemented with 10% fetal calf serum (FBS) from Gibco Co. (Rockville, MD, USA). Dimethyl sulfoxide (DMSO), penicillin streptomycin, and trypsin were purchased from Gibco Co. We analyzed the calcium response of cells in confluency of ∼300 cells/mm2. Changes in cytosolic Ca^2+^ concentration ([Ca^2+^]_i_) were monitored with radiometric Fura-2 fluorescence as described previously [17]. Fluorescence images were recorded at 15-sec intervals with a MicroMAX digital camera (Princeton Instruments Inc., Trenton, NJ). As a positive control for Ca^2+^ stimulation, 10 μM ionomycin (Invitrogen (Grand Island, NE), a Ca^2+^ ionophore (Sigma-Aldrich, St. Louis, MO) or 1 μM ATP (Sigma-Aldrich) was used. Changes of [Ca^2+^]_i_ are presented as Fura-2 fluorescence F340/F380 ratio normalized to that value recorded in cells before stimulation.

### Autoradiography

Kidneys from untreated 8-weeks-old Wistar rats were removed and frozen in isopentane. For radioligand binding displacement experiments, eight consecutive middle transverse kidney cryosections (10 μm/slide) of 6 rats were used to compare potential binding of OT and OT-GKR to V_2_R by autoradiography. Briefly, cryosected tissues were pre-incubated for 15 minutes in Tris Buffer (Tris-HCl 170 mM pH 7.4; MgCl_2_ 5mM) and 0.1% bovine serum albumin (BSA, Thermofisher Gibco Co. Rockville, MD). Slide-mounted sections were then incubated for 180 minutes in the same buffer containing ^125^I-AVP (50 000 cpm,; Dupont-NEN Research Products, Boston, MA). Radioligand binding displacement was determined by co-incubating ^125^I-AVP (PerkinElmer Life and Analytical Sciences Boston) with AVP (Sigma-Aldrich, 10^-6^M), OT (10^-6^M) and OT-GKR (10^-6^M and 10^-8^M). The incubation was followed by 4 x 5-min washes in cold buffer containing 0.1% TritonX (Sigma-Aldrich). Ssections were then rinsed in ice-cold distilled water and dried with a cold air stream. Plaque autoradiography was generated by affixing the slide-mounted section to storage phosphor screen in exposure cassette (Amersham Bioscience, Piscataway, NJ) for 24h and then scanned using Phosphorimager SI scanning (Amersham Bioscience). The radioactivity values of each region of interest were expressed as photo-stimulated luminescence units using Image J thresholding of the area fraction measurement based on histograms. Non-specific binding was determined with the presence of iodated and non-iodated ligand. Specific binding was calculated by subtracting non-specific binding from total binding.

### Intracellular cAMP release in AVP-R2 CHO-K1 Cells

AVP-R2 CHO-K1 cells **(**Eurofins DiscoverX Corporation, San Francisco, CA) were treated with different concentrations of AVP, OT and OT-GKR during 30 minutes at 37°C in one 96-well plate. In one set of cells, AVP (with serial dilutions) was used as antagonist (15 minutes pre-incubation at 37°C) followed by challenging cells with OT-GKR (as agonist at EC_80_ of 2.8x10^-7^ M) during 30 minutes at 37°C. In another set of cells, OT-GKR was used as antagonist followed by challenging cells with AVP as agonist (at EC_80_ of 3.6x10^-9^ M). Changes in intracellular cAMP level in response to agonist stimulation of the GPCR receptor was detected with a chemiluminescent cAMP Hunter eXpress Cat.No: # 95-0150E2CP2M kit from DiscoveRx (www.discoverx.com) [18]. A standard curve of cAMP (in duplicate) was included in the same plate and used to report data in nM cAMP. All data were in the detection limit of the standard curve. The values of cAMP were presented as relative light units (RLU) or they were calculated in nM from the standard curve.

### Molecular docking

Virtual interactions of OT-GKR with OTR and V_2_R were analysed with MolDock software. 3-D models of OT-GKR and OTR and V_2_R were constructed with the Biopolymer module of the SYBYL molecular modeling package (Tripos Associates, St. Louis, MO) as previously validated [19, 20]. The docking scoring function of MolDock computed score grids for dock evaluation and potential binding sites were detected with the grid-based cavity prediction algorithm. The saved conformations for ligand-receptor complexes were subjected to detailed 3-D analysis for interactions at active sites.

### Statistical analyses

All results are expressed as means ± SEM. For dose-related treatment comparisons, paired Student’s *t-*test was used. Multiple group comparisons were made using two-way or one-way ANOVA analysis of variance, followed by an appropriate post hoc analysis. Calculations were performed using GraphPad Prism Software (San Diego, California, USA). A p<0.05 was considered statistically significant.

## Results

### *In vivo* renal effects of OT and OT-GKR

**Fig 1:**
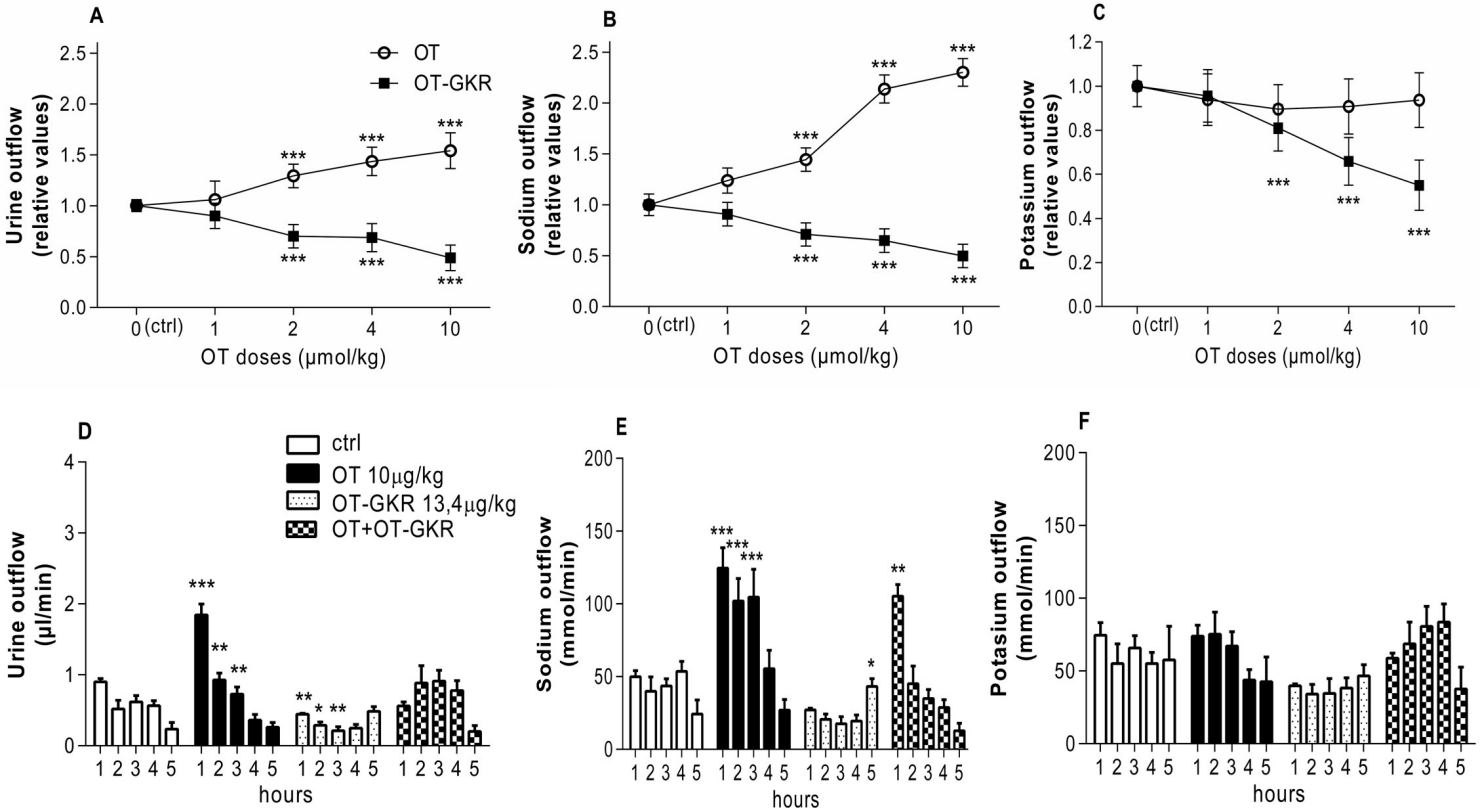
Dose-dependent effects of OT and OT-GKR on urine and electrolyte flow in rat kidney. (A) Equimolar doses of oxytocin (OT) and oxytocin extended form (OT-GKR) were administered intravenously at different concentrations (1, 2, 4 and 10 μmol/kg) to assess urine, (B) sodium and (C) potassium outflow over a period of 5 hours. The effects of equimolar doses of OT and OT-GKR (10 μmol/kg) on (D) urine, (E) sodium and (F) potassium outflow. Both OT and OT-GKR were administered as a single treatment and in combination (OT+OT-GKR). Open circles represent OT and black squares represent OT-GKR. Results are expressed as relative values compared to the control (ctrl) saline injection. Statistical analysis was performed using a one-way ANOVA followed by a Dunnett’s post-hoc test. *: p<0.05, **: p<0.01, ***: p<0.001 compared to the ctrl injection.

To gain an understanding of the mechanisms of action of OT-GKR on kidney function, we first characterized the effects of OT and OT-GKR on diuresis and electrolyte excretion by performing *in vivo* dose-dependent experiments in rats over a period of 5 hours. The effects of these peptides on kidney function, expressed as diuresis, natriuresis, and kaliuresis, are illustrated in Fig 1. Administration of OT increased diuresis (Fig 1A) and natriuresis (Fig 1B) in dose-dependent manner, whereas administration of OT-GKR resulted in dose-dependent inhibition of diuresis (Fig 1A), natriuresis (Fig 1B) and kaliuresis (Fig 1C). The minimal concentrations of 2 μmol/kg were required for induction of these effects by both peptides. The major effects were observed at doses of 10 μmol/kg for both OT and OT-GKR. At these concentrations, OT increased urine outflow by 50% (Fig 1A, p <0.001) and sodium outflow by 130% (Fig 1B, p <0.001) without affecting potassium levels in the urine (Fig 1C). In parallel measurements, OT-GKR decreased urine outflow by 50% (p <0.001), sodium outflow by 50% (p <0.001) and the potassium outflow by 45% (p <0.001).

Fig 1A–1F show a time-course analysis of quantities of urine and electrolytes excreted per hour during a 5 hour after treatment. As presented in Fig 1A, a two-way Anova analysis demonstrated significant main effects of treatment (10 μmol/kg OT or 10 μmol/kg OT-GKR and in combination of both peptides) on diuresis (F1, 400 = 26.70; p <0.001), significant effect of time (F1, 400 = 23.05; p < 0.001) and the interaction of treatment x time (F1, 400 = 12.27; p <0.001). Significant effects of both peptides were observed only from 1 to 3 hours after injection. Our data indicated that diuresis evoked by OT treatment was completely inhibited by co-administration of equimolar concentration of OT-GKR (Fig 1D). Similarly, as presented on Fig 1E, the significant main effects of treatments with both peptides administrated alone were observed on natriuresis (F1, 400 = 34.71; p <0.001), significant effect of time (F1, 400 = 13.10; p < 0.001) and the interaction of treatment x time (F1, 400 = 6.23; p <0.001). It can be observed that a potent natriuretic effect induced during 3 hours after OT injection was reduced by co-treatment with OT-GKR in samples collected at hour 2 and 3 but not changed in the sample obtained at the first hour. Kaliuresis was affected by peptide treatment (F1, 400 = 5.52; p <0.001), but not affected by time (F1, 400 = 1.13; p = 0.34) and interaction of both factors (F1, 400 = 1.11; p = 0.35), (Fig 1F).

### The effects of OT and OT-GKR on diuresis and electrolyte excretion in the presence of OTR and V_2_R antagonists

**Fig 2:**
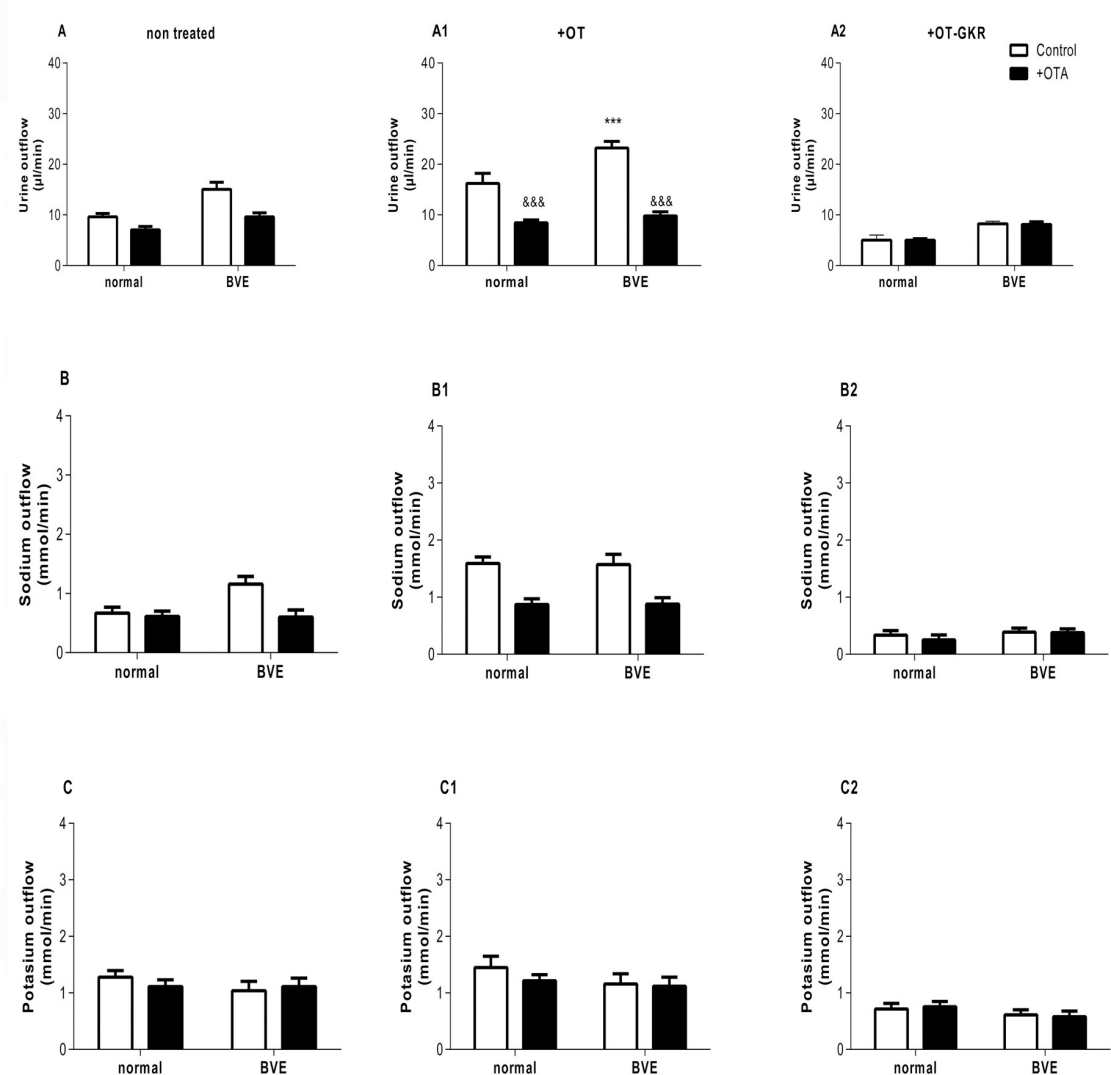
The effects of oxytocin (OT) and oxytocin extended form (OT-GKR) on diuresis, natriuresis and kaliuresis in the presence of an oxytocin receptor antagonist (OTA). (A-A2) diuresis, (B-B2) natriuresis and (C-C2) kaliuresis. Measurement of urine and electrolyte outflow was performed following a normal volume injection (0.1 mL) and with blood volume expansion (5 mL, BVE). Statistical analysis was performed using a two-way ANOVA followed by a Bonferroni post-hoc test. *: p<0.05, **: p<0.01, ***: p<0.001 compared to the ctrl injection.

We performed experiments with OTR and V_2_R antagonists to disclose parallel diuretic, natriuretic and kaliuretic actions involving these receptors in the body. The experiments were performed under normal conditions after iv injections of a small vehicle volume (0.1 mL) and after blood volume expansion (BVE, 5 mL). A volume of 5 mL was selected based on our earlier study indicating that OT levels in the plasma are increased and diuresis, natriuresis and ANP release from the heart are stimulated [7]. The effects of OT or OT-GKR in the presence of specific antagonist for OTR (OTA) is illustrated in Fig 2. As presented in Fig 2A, analysis of diuresis induced by BVE demonstrated significant main effects of treatment with 10 nM OTA (F1, 44 = 18.67; p <0.001), significant effect of volume expansion (F1, 44 = 18.39; p < 0.001) and lack of interaction of treatment x BVE (p = 0.13). This interaction reached significance when all animal groups received 10 nM OT (Fig 2A1; F1,44 = 4.78; p = 0.034). Post-hoc analysis of data revealed that the observed increase in diuresis in BVE rats was further elevated after OT administration (p<0.001). Treatment with OTA inhibited OT-stimulated diuresis to control and BVE levels. When rats received 10 nM OT-GKR, diuresis in control and BVE groups was not changed after OTA co-administration (Fig 2A2; F1,44 = 0.91; p = 0.91). However, some effect of BVE on diuresis was still significant (F1,44 = 0.01; p<0.001).

**Fig 3:**
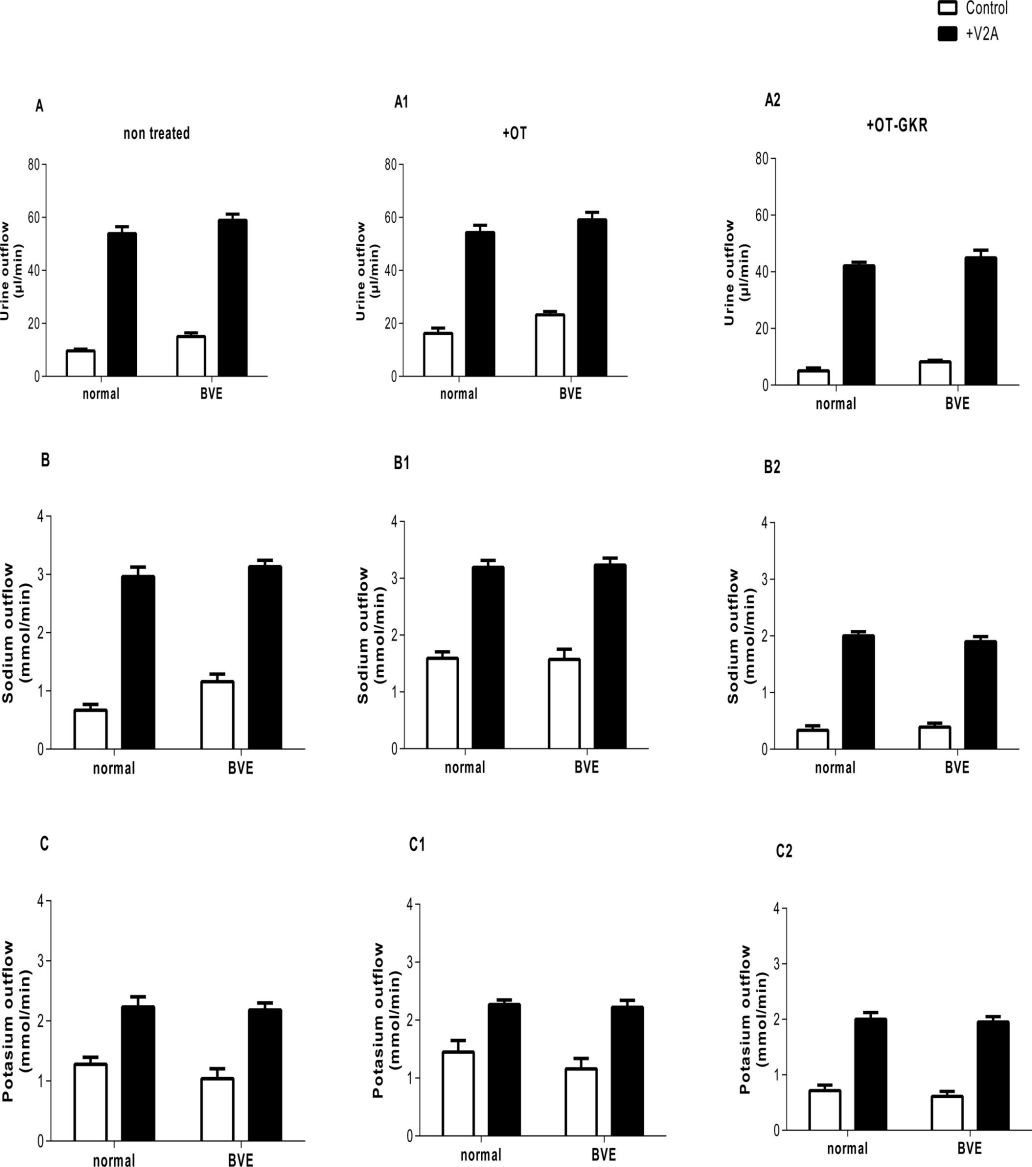
The effects of oxytocin (OT) and oxytocin extended form (OT-GKR) on diuresis, natriuresis and kaliuresis in the presence of a vasopressin receptor 2 antagonist (V2A). (A-A2) diuresis, (B-B2), natriuresis and (C-C2) kaliuresis. Measurement of urine and electrolyte outflow was performed following a normal volume injection (0.1 mL) and with blood volume expansion (5 mL, BVE). Statistical analysis was performed using a two-way ANOVA.

Differences in natriuresis were also observed in rats subjected to BVE and treatment with OTA, OT and OT-GKR (Fig 2B–2B2). Natriuresis was significantly increased by BVE. However, in contrast to the effects of OT on diuresis, natriuresis was not enhanced in the presence of OT (Fig 2B1). Interestingly, when rats received OT-GKR, natriuresis was abnormally low in all groups and the effect of BVE was not significant (Fig 3B2, F1,44 = 0.38; p = 0.24). Kaliuresis was not regulated in these experimental conditions (Fig 2C–2C2)

Similar experiments with application of the V_2_R antagonist (V_2_A) are illustrated on Fig 3. Treatment with V_2_A alone resulted in significant diuresis associated with robust effects on sodium and potassium outflow (Fig 3). Thus, the relatively low diuretic and natriuretic effects of OT observed in the absence of V_2_A were not apparent when V_2_R was blocked (Fig 3A1–3B1). However, the diuretic and natriuretic effects of V_2_A were in part inhibited when rats received OT-GKR (Fig 3A2–3B2). Because V_2_R inhibition induced a powerful kaliuresis, low inhibition of kaliuresis by OT-GKR was undetected in control or BVE conditions. Diuretic and natriuretic effects of V_2_A were partially inhibited by OT-GKR co-administration (Fig 3A2–3B2). Because V_2_R antagonism induced a potent kaliuretic response, modest inhibition of kaliuresis by OT-GKR treatment was undetected under control and BVE conditions (Fig 3C2).

### *In vitro* competition binding autoradiography of OT-GKR with AVP and OT

**Fig 4:**
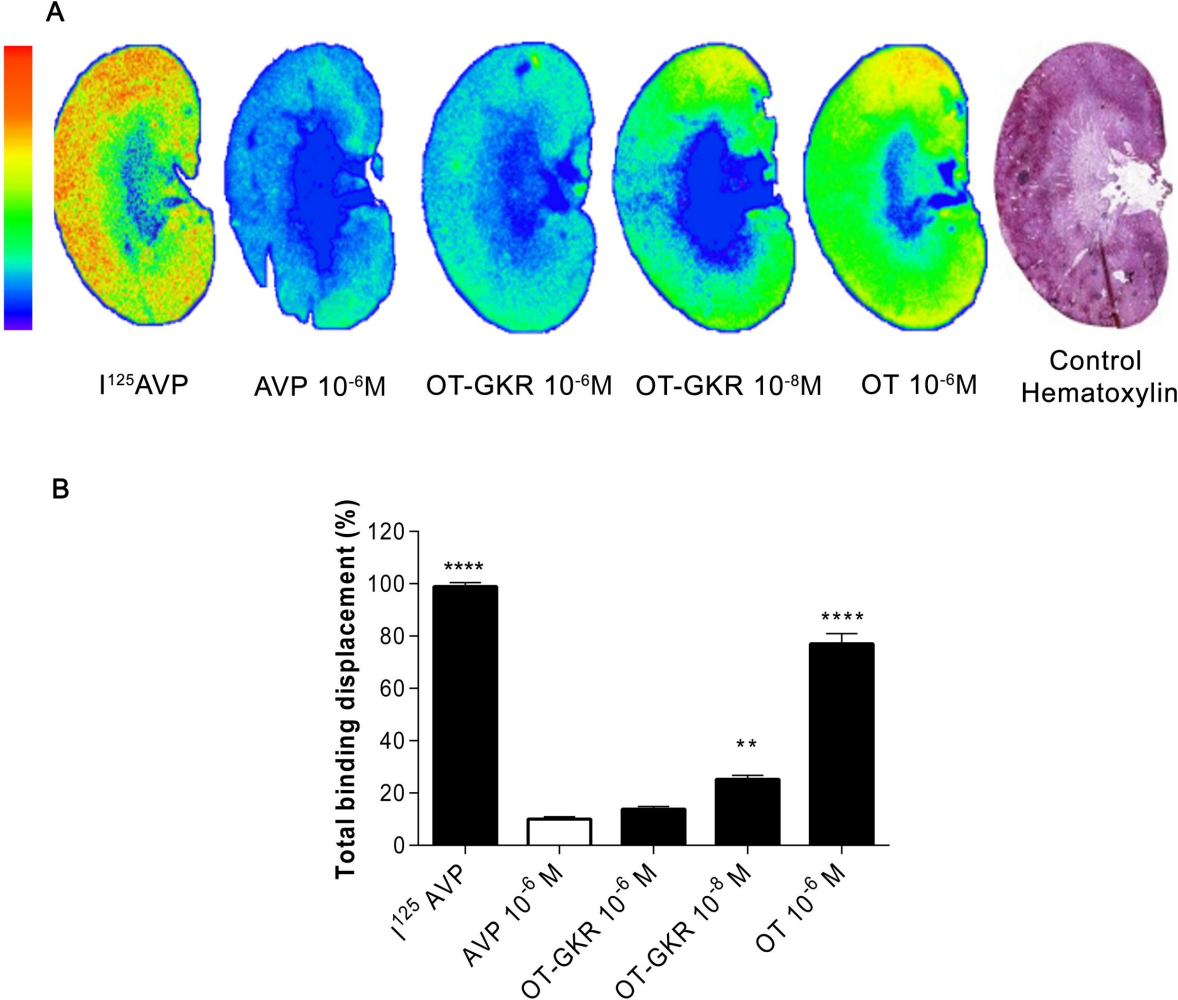
Autoradiography kidney representations of AVP, OT-GKR and OT in competition with I^125^AVP. (A). Representative images of autoradiography registered by Phosphorimager. (B) The competitive binding efficiency of AVP, OT-GKR, OT, with I^125^AVP. Statistical analysis: One-way ANOVA with a Dunnett’s post-test. *: p<0.05, **: p<0.01, ***: p<0.001 *vs* total binding displacement.

Autoradiography was used to disclose the competitive binding of OT-GKR or OT with AVP binding sites on kidney sections (Fig 4). The binding sites of AVP were localized in the cortex and outer medullary area of the kidney. The photo-stimulated luminescence displaced by 10^-6^M concentration of corresponding cold peptide from kidney sections incubated with ^125^I-AVP were considered arbitrary as 100%. Fig 4. Shows that OT-GKR at the concentrations of 10^-6^M and 10^-8^M potently displaced ^125^I-AVP radioactivity bound to rat kidney sections (85.9 ± 12% and 74.5 ± 19.7%, respectively). These displacement values were not different to the displacement results obtained with cold AVP 10^-6^M. The displacement of ^125^I-AVP from kidney sections was significantly lower by cold OT (22.0 ± 9.7; p<0.001).

### Computerized docking analysis

**Fig 5:**
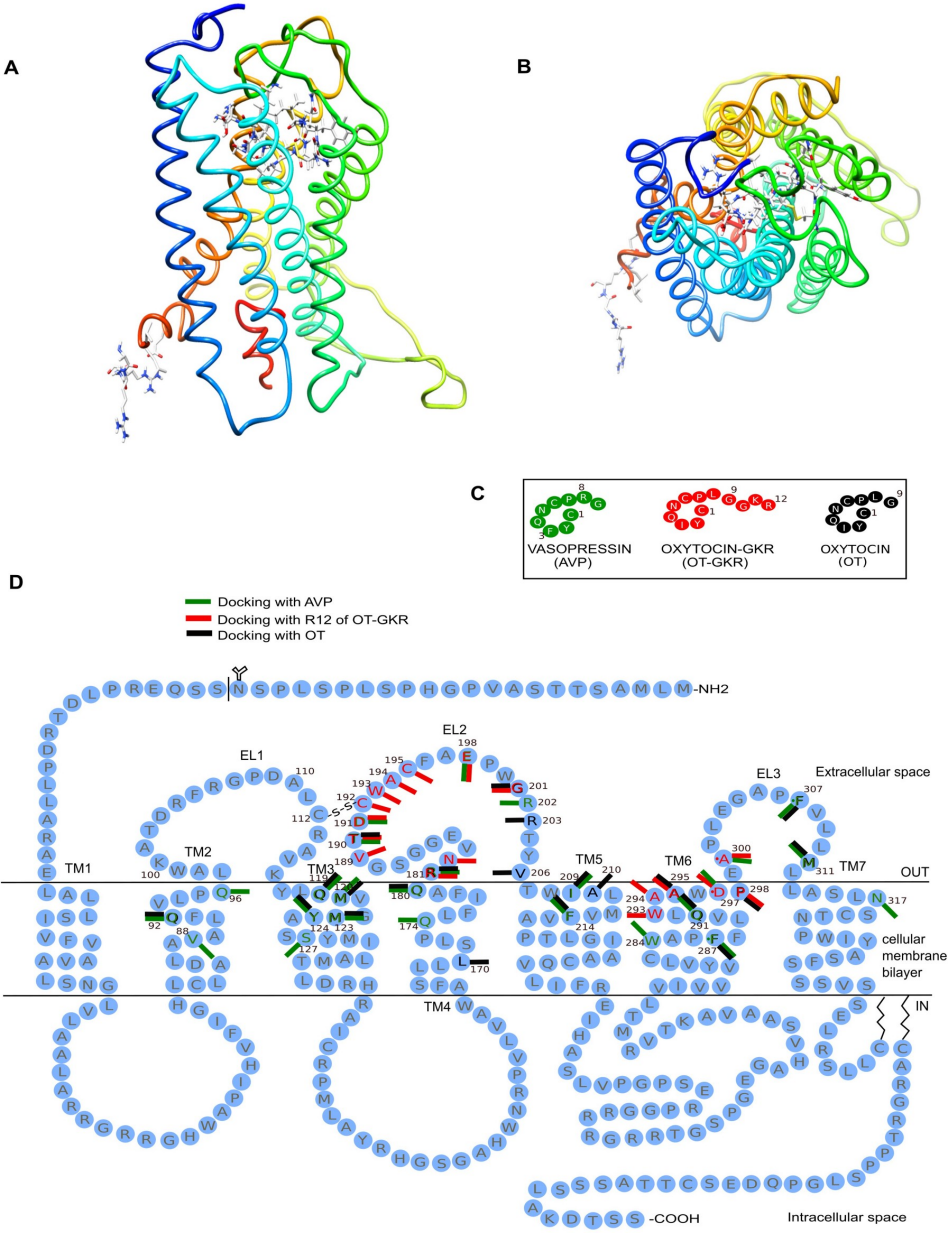
Schematic model of human vasopressin receptor (V_2_R) binding with oxytocin (OT), oxytocin extended form (OT-GKR), and arginine vasopressin (AVP). (A) The front upright view position (side view) of the receptor structure with OT. (B) The top intracellular view (i.e. rotation by 90^o^ out of plane) of OT-GKR inside the OTR binding site.(C) Conformational view of AVP, OT-GKR, and OT and (D) schematic model of human vasopressin V_2_R binding with AVP, OT-GKR and OT. The amino acid residues in black circles have been proposed as OT docking sites, the red bars represent docking sites of OT-GKR and the green bars represent docking sites of AVP. Amino acid residues are identified by a 1-letter code in Table 1.

We have introduced computerized 3-D analysis of molecular docking at the receptor sites to study the potential interaction between a OT-GKR (also OT and AVP) and a V_2_R protein at the atomic level. This analysis will allow to characterize the behavior of tested molecules in the binding site of target proteins as well as to elucidate fundamental biochemical processes[21]. The analysis revealed significant binding energies of OT-GKR to V_2_R. Fig 5 illustrates the docking human V_2_R with OT-GKR and OT-modeled molecules in front upright view (Fig 5A) and a view from the extracellular side (Fig 5B). Three conformations for OT-GKR were analyzed and six related V_2_R- OT-GKR best energy-relaxed complexes were selected. Possible hydrophobic and electrostatic interaction points in dynamic complexes of these molecules were indicated by estimated binding affinity energies of -6.6±0.4 kJ/mol for OT-GKR and -11,146±1.52 kJ/mol for AVP. Using distance criteria, the program identified receptor amino acid residues interacting with ligands. The essential hydrogen bond and strong electrostatic interactions between both OT molecules and the receptors were characterized by visual inspection. The results are reported in Fig 5D and Table 1. Several amino acid residues have been proposed to interact with AVP and OT-GKR in the V_2_R model; red bars represent docking with OT-GKR, green bars indicate docking with the molecular model of AVP, and the black bars show docking with the OT molecule (Fig 5D). Docking to V_2_R in positions D297, R181, T190, D191, E198 and A300 were noted for AVP as well as for OT-GKR molecules (Table 1). OT-GKR binding sites were concentrated in extracellular loop 2 (EL2) of V_2_R. The OT-GKR was bound exclusively in positions of W293, A294, D297, N182, V189, C192, W193, A194 and C195. These docking positions constituted 25% of all observed docking sites of OT-GKR. OT-GKR interactions with V_2_R were observed mainly at terminal part of molecule corresponding to the arginine in position 12 (Fig 5D).

**Table 1:** List of the V_2_R residues interacting with arginine-vasopressin (AVP), oxytocin (OT) and carboxy-extended form of oxytocin–OT-GKR.

	AVP	OT-GKR	OT
TM2	V88	-	-
	Q92	-	Q92
	Q96	-	-
TM3	Q119	-	Q119
	M120	-	M120
	M123	-	M123
	Y124	-	Y124
	S127	-	-
TM4	-	-	L170
	Q174	-	-
	Q180	-	Q180
TM5	I209	-	I209
	-	-	A210
	F214	-	F214
TM6	W284	-	-
	F287	*-*	F287
	Q291	-	Q291
	-	W293	-
	-	A294	-
	-	A295	A295
	D297	D297	-
	-	P298	P298
TM7	N317	-	-
EL2	R181	R181	R181
	-	N182	-
	-	V189	-
	T190	T190	T190
	D191	D191	-
	-	C192	-
	-	W193	-
	-	A194	-
	-	C195	-
	E198	E198	-
	-	G201	G201
	R202	-	-
	-	-	R203
	-	-	V206
EL3	A300	A300	-
	E307	*-*	E307
	M311	-	M311

### The effects of OT and OT-GKR on intracellular Fura-2-based calcium measurements in H9c2 cardiac cells

**Fig 6:**
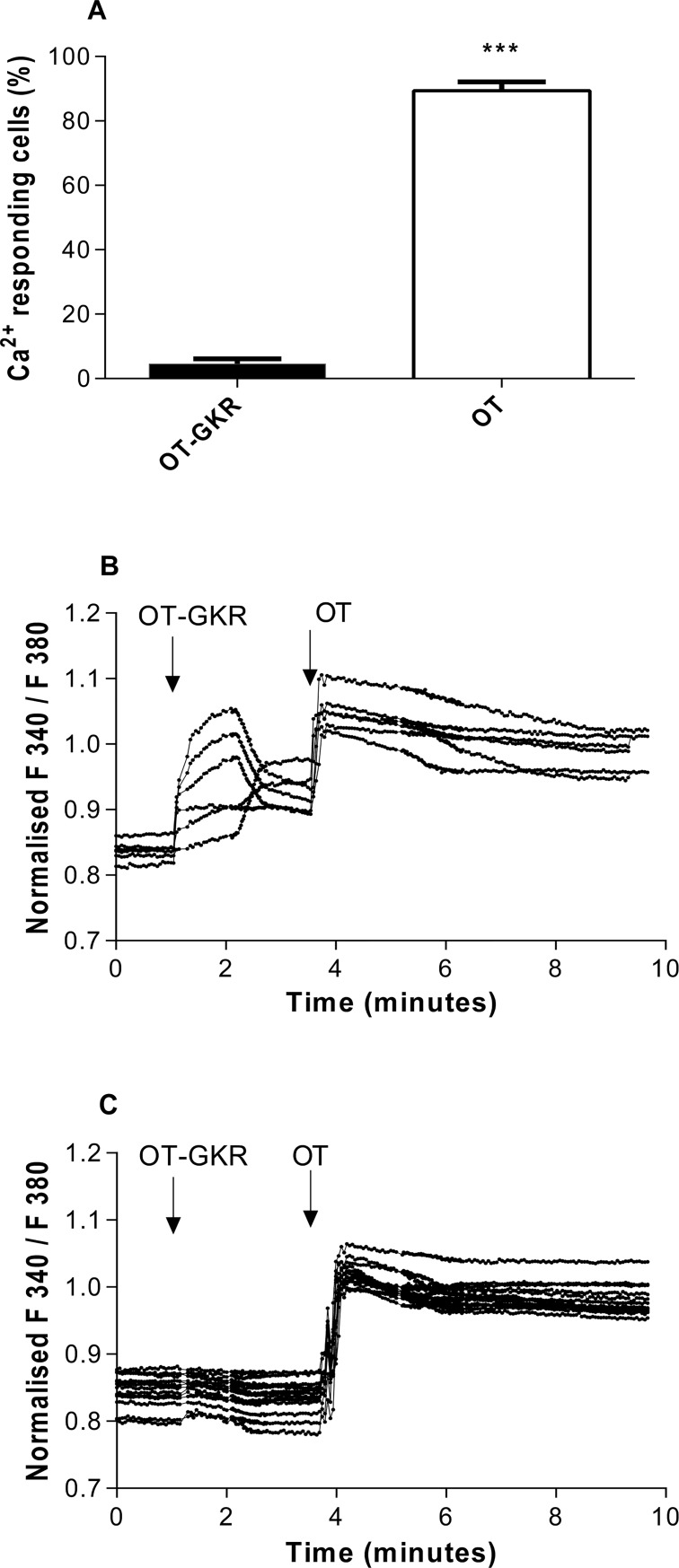
The effects of oxytocin (OT) and oxytocin extended form (OT-GKR) on intracellular calcium levels measured in Fura-2-loaded H9c2 cells. (A). Percentage of calcium-responsive cells counted per microscopic field after treatment with equimolar concentrations (10^−6^ M) of OT and OT-GKR. (B) Kinetics of intracellular calcium levels normalized as the F340/F380 ratio in cells responding to OT-GKR and (C) in cells resistant to OT-GKR Data were obtained from 7 separate experiments. For (A), statistical analysis was performed using a Students t-test. Data are presented as mean ± SEM. ***: p<0.001, compared to OT-GKR-treated cells.

In most cells studied, OT exerts effects *via* binding to specific receptors with subsequent stimulation of Ca^2+^ messenger systems [22]. Previous Ca^2+^ mobilization studies in D3 cells showed functional OT-GKR and OT activity in embryonic stem cells[23]. The activity of OT-GKR and OT in H9c2 cardiac cells was recorded by cytosolic Ca^2+^ concentration ([Ca^2+^]_i_) in single-cell photometry which allows to disclose the spatial changes in the kinetics and amplitude of Ca_i_^2+^ transients in response to various peptides. Fig 6A shows the percentage of [Ca^2+^]_i_ positive cells responding to the concentration of 10^−6^ M OT-GKR and 10^−6^ M OT resulting in the maximal [Ca^2+^]_i_ stimulation by both peptides. Only 8.66 ± 1.36% cells (n = 6 independent experiments) presented a detectable increase in [Ca2+]_i_ upon stimulation with 10^−6^ M OT-GKR whereas 88.05 ± 1.13% cells responded with 10^−6^ M OT (Fig 6A). The number of cells activated by OT was close to the number of cells responding to 1 μM ATP, used as a positive control. In addition, the amplitude of the [Ca^2+^]_i_ response in single cells was significantly lower in cells stimulated with OT-GKR (Fig 6B) compared to cells activated by OT (Fig 6C). Interestingly, treatment of cells with OT-GKR did not influence the [Ca^2+^]i response to subsequent treatment with OT (Fig 6B). The results of these experiments suggest that OT and OT-GKR may be involved in regulating [Ca^2+^]_i_ levels by different pathways.

### The effects of OT, OT-GKR, and AVP on intracellular cAMP in AVP-R2 CHO-K1 cells

**Fig 7:**
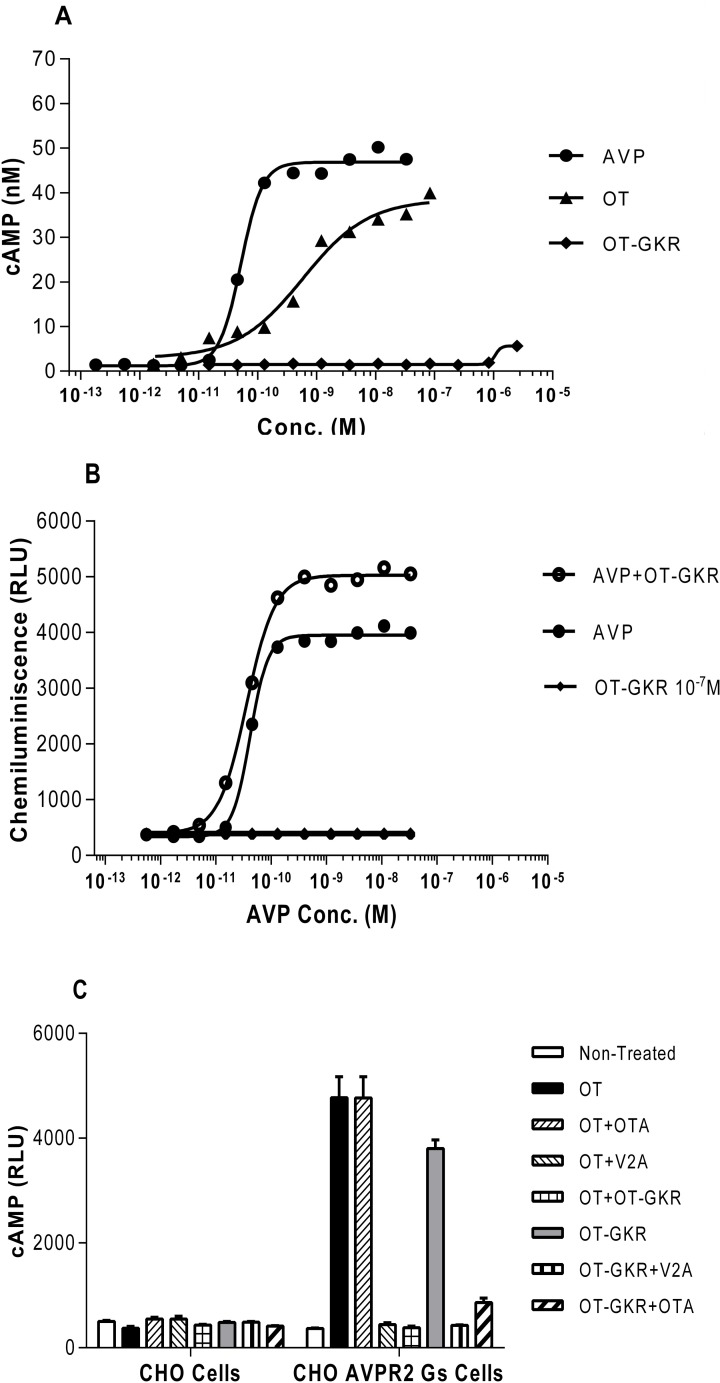
The effects of arginine vasopressin (AVP), oxytocin (OT), and oxytocin extended form (OT-GKR) on intracellular cAMP levels in AVP-R2 CHO-K1 cells. (A) Release of cAMP from AVP-R2 CHO-K1 cells in response to increasing concentrations of AVP, OT, and OT-GKR. (B) Release of cAMP from AVP-R2 CHO-K1 cells treated with AVP as antagonist and in combination with OT-GKR used as agonist. OT-GKR was added in AVP-R2 CHO-K1 cells at the concentration of 10^-7^M. (C) Release of cAMP from control CHO cells and AVP-R2 CHO-K1 cells containing the vasopressin receptor 2 (V_2_R). Cells were stimulated with OT and OT-GKR in the presence of the V_2_R antagonist (V2A, 10^−5^ M) or the oxytocin receptor antagonist (OTA, 10^−6^ M).

As illustrated in Fig 7A, AVP and OT potently stimulated cAMP release from V_2_AVP-R2 CHO-K1 cells overexpressing V_2_R whereas OT-GKR had no measurable effect (Fig 7A). Corresponding EC50 values estimated from standard curves were 4.2e-011 for AVP, 3.2e-010 for OT and 1.1e-006 for OT-GKR. AVP measured as antagonist compared to OT-GKR (2.8 x 10^−7^ M) used as agonist induced a greater increase in cAMP (EC50 = 3.6 e-011) levels than AVP alone (4.2e-011) (Fig 7B). The OT-stimulated effect on cAMP production was reduced in the presence of the V_2_R antagonist (10^−5^ M V_2_A) but not by the specific OTR antagonist (10^−6^ M OTA) (Fig 7C). On the other hand, the combination of OT and OT-GKR in concentrations adjusted to the EC80 prevented the increases in cAMP release. The OT-GKR effect on cAMP release was also completely reduced in the presence of V_2_A and partially inhibited in the presence of OTA.

## Discussion

We have demonstrated that administration of precursor OT peptide, OT-GKR, induces anti-diuretic, -natriuretic, -kaliuretic effects in rat kidney. These actions are opposite to effects of the amidated OT nonapeptide. After combined injection of OT and OT-GKR, our results indicate that OT-GKR competes with low concentration of OT in control and blood volume expanded conditions. OT-GKR dose-dependently inhibited OT-evoked diuresis but not natriuresis. Injection of OT antagonist OTA resulted in inhibition of diuresis and natriuresis exerted by OT, whereas OT-GKR administration inhibited diuresis even in the presence of OTA. This suggests that OT-GKR is acting by the V_2_R subtype in the kidney. This effect was supported by the observation that diuresis induced after V_2_ receptor antagonist injection was, in part, reduced by OT-GKR co-administration. Autoradiography revealed specific binding of OT-GKR to AVP-specific binding sites in rat kidney. Molecular docking modeled by computer software revealed the binding sites for OT-GKR on the structure of V_2_R and OTR. In cells overexpressing V_2_R, OT-GKR alone weakly induced cAMP in cells overexpressing V_2_R, but enhanced cAMP release when combined with AVP. In contrast, OT-GKR inhibited cAMP release induced by OT treatment. Taken together, our results indicate that OT-GKR modulates the function of OTR and V_2_R in kidney and may a role in regulation water and electrolyte homeostasis in conditions of enhanced OT-GKR levels in the circulation. This effect might have important implications in estrogen replacement therapy, and postpartum and postnatal diuresis associated with enhanced concentrations of extended OT molecules in the circulation [24].

### Functional effects of OT and OT-GKR on kidney

In our previous studies, we reported that the OT-GKR potently increased the spontaneous formation of cardiomyocytes in embryonic stem cells[20],[23] and augmented glucose uptake in newborn rat cardiomyocytes above the level stimulated by OT [25]. Elevation in the plasma levels of OT prohormones is associated with periods characterized by dramatic changes in electrolyte and water homeostasis such as with labor [26] and adaptation to extra-uterine life [16]. Mitchell et al reported that at parturition the concentrations of extended forms of OT in uterine tissue were 5- to 30-fold greater than those of OT, and both increased progressively throughout the late gestation period [14]. However, OT-GKR binds weakly to the OTR in uterine tissue [24] although the number of OTR is also elevated in uterus during parturition. It was hypothesized that the extended forms of OT have little biological activity in the uterus during pregnancy but would complete with OT for the OTR and as such might play a role in the regulation of labor [14]. We studied whether this hypothesis can be extended to kidney function by analyzing the effects of OT-GKR in combination with OT under normal conditions and with BVE. Our results show that OT-GKR opposes the diuretic and natriuretic effects evoked by iv administration of low doses of OT. Low doses of OT were selected in order to prevent ant potential effect of V_2_R activation by OT. Urine analysis was performed shortly after i.v. administration to avoid receptor desensitization and thus increasing their sensitivity to agonists and antagonists. Initially, i.v. injections were performed with a small volume to not influence total body fluid volume and hemodynamics. In these conditions, OT caused a dose-dependent increase of diuresis and natriuresis with involvement of OTR since OTA inhibited these effects. This observation is consistent with data of Verbalis et al.[6] who demonstrated that OT-induced natriuresis was inhibited by OTA treatment but not by AVP antagonist. In our study, treatment with OT-GKR caused a significant dose-dependent decrease of diuresis, natriuresis and kaliuresis. The overall effect of OT-GKR mimicked the actions of AVP. Blocking OTR did not reverse the renal effects induced by OT-GKR whereas OT-GKR partially blocked the diuretic effect of the V_2_A. Therefore, these results are consistent with our hypothesis that OT-GKR induces renal effect in kidney by action on V_2_R.

### Effects of OT and OT-GKR on kidney with blood volume expansion

Our experiments suggest an important contribution of the OT system in water and electrolyte balance following blood volume expansion. BVE increased diuresis and natriuresis that was likely attributed to hypothalamic OT and cardiac ANP release in the circulation [8]. ANP is also secreted with normal blood volume conditions following activate of OTR by OT [27]. Sodium excretion induced by OT treatment was similar under normal and with BVE and was higher than natriuresis induced by BVE alone. However, we observed a synergistic effect of BVE and OT on diuresis. The single injection of OTA decreased urine outflow, but not sodium outflow under normal conditions. This can be explained by blockade of OTR because the antagonist has negligible effects on V_2_R [28]. Moreover, in this study we presented that OTA did not interfere with OT-GKR effects on inhibition of diuresis and natriuresis. In addition, OTA treatment completely inhibited diuresis and natriuresis induced by OT under normal conditions. This observation is consistent with a previous report which demonstrated that OTA treatment of anaesthetized rats resulted in an abrupt and sustained reduction in Na^+^ excretion accompanied by a significant reduction in urine flow rate [29]. These results indicate that OTR in kidney participate in the endogenous control of natriuresis and water reabsorption possibly via endogenous nitric oxide regulation in the macula densa [7]. The dissociation of water and sodium excretion suggests separate mechanisms for water and electrolyte control and can be explained by the increase of ANP release from the heart in response to BVE and OT administration [8, 27]; this effect potentially inhibited by OTA co-administration [27]. Under BVE conditions, administration of OT-GKR inhibited diuresis and decreased natriuresis in BVE rats more effectively than under normal conditions. The fact that OT-GKR significantly reduces the powerful diuretic and natriuretic effects of V_2_A indicates that V_2_R is targeted by OT-GKR.

### Kidney receptors and OT-GKR docking

The antidiuretic effect of AVP is mediated by the V_2_R. The receptors for OT and AVP share structural homology. As OT can also bind to the AVP receptor but with lower affinity [1], several studies have suggested the possibility that the anti-diuretic action of OT is mediated via V_2_R [9, 10]. Although both OTR and V_2_R transcripts were identified found in rat collecting duct by RT-PCR, only the V_2_R antagonist can could block the hydro-osmotic response to 200 pM OT, implying that the actions of OT occur by the V_2_R [30]. In our previous report, computerized analysis demonstrated that OT-GKR and OT have both similar and different docking sites inside the OTR and V_1_ receptor [20]. As cell differentiation and glucose uptake occur by activation of OTR and V_1_ receptors [25, 31], this partly explains that OT-GKR, compared to OT, has a greater ability to stimulate cardiomyocyte differentiation in stem cells [20, 23, 32] as well as potential to augment glucose uptake in cardiomyocytes [25]. On the other hand, OT-GKR, just as OT, stimulates the production of ANP in rat cardiomyocytes by mechanisms involving OTR and V_1_R and possibly Ca^+2^ [33]. In this study, we found that OT-GKR stimulated Ca^+2^ mobilization in some cells but this effect was further potentiated if the same cells were subsequently treated with the amidated form of OT. This suggests that Ca^+2^ induced by OT-GKR and OT may be mediated by different pathways. Presently, we performed computerized docking analysis to assess the relationship between OT-GKR and V_2_R. Interactions between V_2_R residues and four OT-GKR amino acids, namely Ile-3, Leu-8, Tyr-2 and Arg-12 were observed in V_2_R-OT-GKR complexes. The molecular docking of OT and OT-GKR showed that while both peptides interacted with V_2_R with significant binding energies, the binding pocket for OT-GKR displayed differences compared to binding of AVP and OT. Interestingly, the specific role in binding of OT-GKR molecule may involve arginine (R) in the 12^th^ position. The amidated form of OT does not contain arginine, but AVP does at the 8^th^ position, which can partly explain the shared effect of OT-GKR and AVP. OT-GKR interacts with several sites in seven transmembrane domains of V_2_R. Importantly, the OT-GKR binding sites were abundant in extracellular loop 2 of V_2_R. This region seems to play important role in activation because it is involved in direct binding of the ligand, ligand recognition, and ligand entry [34]. Using bioinformatics analysis data, Sebti et al. [35] recently demonstrated that A300 and D297 of the V_2_R were involved in receptor binding with AVP. They induced an Ala300Glu and Asp297Tyr mutation of V_2_R which led to a low cAMP production. These V_2_R residues were also found in our study as targets of OT-GKR. Based on these observations, we suggest that AVP-like docking into V_2_R present in collecting duct contributes to the anti-diuretic and anti-natriuretic effects of OT-GKR.

### Stimulation of cAMP in CHO cells overexpressing V_2_R

V_2_R located at the basolateral membrane of collecting duct cells activate various adenylate cyclases (AC), which in turn increases cAMP levels and stimulates protein kinase A. AVP is the major GPCR responsible for cAMP generation in isolated collecting ducts [36]. cAMP is produced by multiple isoforms of AC and the roles of individual AC isoforms in water and electrolyte homeostasis are not well-understood. Studies have demonstrated that AC6 mRNA is the most highly expressed AC isoform in the inner medulla. Further, AC6 is inversely correlated with fluid intake and determines AVP-induced cAMP formation in the inner medulla [37]. In our study, receptor regulation of cAMP signaling was explored in V_2_R transfected CHO cells, which predominantly express AC6 [38], and therefore mimics regulation of V_2_R signaling in the kidney. The kidneys are continuously exposed to the tonic action of AVP to avoid dehydration. Therefore, to explain the effect of terminally extended OT molecules on water and electrolyte balance in the rat we should consider our observation that their abundance increases the AVP-mediated cAMP signaling in the kidney. However, OT and OT-GKR combined in high concentrations (EC80) inhibited cAMP release from V_2_R transfected CHO cells. Presently, we have no explanation of this effect since both peptides when added alone stimulated cAMP from these cells. Assuming differences in V_2_R docking of these peptides we can only speculate that receptor conformation in response to combination of OT and OT-GKR results in inhibition in cAMP production in cells.

## Study limitations

The results of our current study are relatively modest in terms of evaluating natriuresis mechanistically. We understand that focusing on OT-GKR- V_2_R -cAMP interactions as presented in this paper represents a minor yet important aspect of oxytocin research. More efforts should be made to measure or infuse hormones such as ANP, AVP, and Ang II, which would allow to disclose the effect of hormones or treatment of interest to be queried. Experiments performed on cells derived from the renal collecting duct co-expressing V_2_R, aquaporin 2 and ENAC may more accurately reflect the signaling pathway in kidney *vs*. what is observed in V_2_R transduced CHO cells. The same cells possess specific receptors for mediators which have been shown to act specifically on cAMP production such as prostaglandins, dopamine, bradykinin, and endothelin which by secondary actions may influence sodium balance rather than water homeostasis [39]. Therefore, it is also possible that OT-GKR as well as OT can affect kidney function during deficiency of functional V_2_R. The possibility also exists that OT-GKR and OT can affect kidney function in nephrogenic diabetes insipidus predominantly related to dysfunctional renal V_2_R.

## Conclusions

OT-GKR is an endogenous prohormone peptide that competes for V_2_R binding to induce anti-diuretic, -natriuretic, -kaliuretic effects in rat kidney. This has a significant physiological importance because elevated levels of elevation OT prohormones in the circulation is associated with dramatic changes of salt and body homeostasis observed in women during labor, in newborn infants, and in women receiving estrogen replacement therapy [16, 26].
